# A Long Fast

**Published:** 1898-02

**Authors:** 


A LONG FAST.—The *Veterinary Journal* reports a case of prolonged starvation, for thirty-eight days, in a bitch which was accidentally buried under straw. She was very fat when lost, but when found was nothing more than skin and bone. Very little excrement was found. She was placed on a milk-diet and recovered.

